# CREB5 Inhibits Neuronal Ferroptosis via Transactivating ApoL6 to Regulate Lipid Droplet Metabolism After Spinal Cord Injury

**DOI:** 10.1002/cns.70783

**Published:** 2026-02-17

**Authors:** Xiaolong Xi, Zhensen Chen, Chaojun Wang, Fei Wang, Xuedong Sun

**Affiliations:** ^1^ Department of Surgical Critical Care Medicine Shaoxing People's Hospital Shaoxing Zhejiang China; ^2^ Department of Orthopedics Shaoxing People's Hospital Shaoxing Zhejiang China; ^3^ Department of Surgical Critical Care Medicine The First Affiliated Hospital, Shaoxing University Shaoxing Zhejiang China

**Keywords:** ApoL6, CREB5, ferroptosis, neurons, spinal cord injury

## Abstract

**Background:**

After spinal cord injury (SCI), neuronal lipid peroxidation and excessive production of reactive oxygen species (ROS) induced by secondary injury exacerbate ferroptosis, impeding regenerative repair and functional recovery in mice. Thus, clarifying the molecular and cellular mechanisms underlying the inhibition of neuronal ferroptosis post‐SCI is crucial.

**Methods:**

Single‐cell RNA sequencing (scRNA‐seq) and single‐cell assay for transposase‐accessible chromatin sequencing (scATAC‐seq) were used to analyze changes in the transcription factor CREB5 post‐SCI. Combined with in vitro (primary neuron experiments) and in vivo (mouse SCI model) studies, CREB5 was knocked down/overexpressed, and ApoL6 was overexpressed. Indicators related to neuronal ferroptosis (ROS, lipid peroxidation, free fatty acids, etc.) and functional recovery in mice were detected.

**Results:**

After SCI, the transcriptional activity of the transcription factor CREB5 is enhanced, and its expression level first increases and then decreases. Mechanistically, CREB5 inhibits the decomposition of neuronal lipid droplets (LDs) by enhancing the transcriptional activity of the lipolysis‐related protein ApoL6, reducing the release of free fatty acids (FFA) and fatty acid oxidation (FAO), thereby decreasing ROS generation and lipid peroxidation, and ultimately inhibiting neuronal ferroptosis. In vitro experiments showed that CREB5 knockdown exacerbates neuronal death and inhibits axonal growth; in vivo experiments demonstrated that CREB5 knockdown hinders axonal growth and functional recovery in mice post‐SCI, while ApoL6 overexpression partially reverses these impairments.

**Conclusions:**

CREB5 maintains the balance of neuronal lipid droplet metabolism by regulating ApoL6 and serves as a potential therapeutic target for inhibiting neuronal ferroptosis after SCI.

## Graphical abstract

After spinal cord injury, neuronal CREB5 exhibits a biphasic trend of increased then decreased transcriptional activity and expression. By transactivating ApoL6, CREB5 inhibits lipid droplet catabolism, reduces reactive oxygen species and lipid peroxidation to suppress ferroptosis, thereby promoting axonal growth and functional recovery in mice.

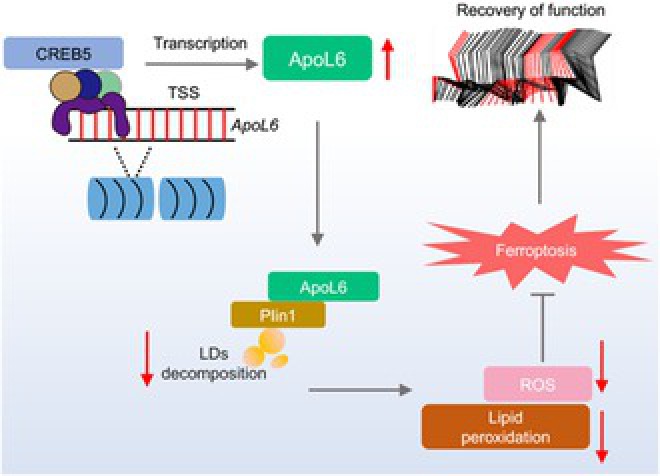

## 1 Introduction

Spinal cord injury (SCI) is a highly disabling trauma that often results in severe motor and sensory dysfunction, imposing a heavy economic burden on society and families [1]. A series of secondary injuries caused by SCI, particularly in the acute and subacute phases post‐injury, can lead to excessive production of ROS and lipid peroxidation in neurons [2, 3, 4]. This, in turn, increases the sensitivity of neurons to ferroptosis, reduces neuronal viability, exacerbates neuronal death, and ultimately hinders subsequent regeneration and functional recovery [4]. Therefore, it is crucial to identify the molecular and cellular mechanisms following SCI to alleviate neuronal ferroptosis.

The full name of TF CREB5 is cAMP response element‐binding protein 5, which is a TF encoding zinc finger structure (C2H2 type) and basic leucine zipper (bZIP) domain, belonging to the CREB/ATF family. Studies have found that CREB5 can serve as a ferroptosis‐related biomarker in alopecia areata [5]. In addition, in Alzheimer's disease, miR‐32,533 regulates oxidation and neuroinflammation by targeting CREB5 [6]. However, research on CREB5 inhibiting neuronal ferroptosis in SCI has not been reported.

Ferroptosis has been identified as one of the forms of programmed cell death after SCI, among which the accumulation of lipid peroxidation products and excessive production of ROS are important characteristics of ferroptosis [7, 8]. Studies have found that the lipid droplet‐associated protein ApoL6 can inhibit LD lipolysis through direct interaction with Plin1 [9]. However, after SCI, neurons, due to insufficient energy supply, will increase the utilization of energy substrates other than glucose, thereby accelerating fatty acid decomposition [10, 11]. It is worth noting that neurons are extremely sensitive to elevated levels of FAO, and excessively high FAO can lead to increased ROS levels and lipid peroxidation [12, 13]. In addition, excessive FFA may enter non‐oxidative metabolic pathways, promoting the production of excessive neurotoxic ceramides, which in turn damage neurons [14].

In this study, through scRNA‐seq combined with scATAC‐seq, we found that both the transcriptional activity and expression level of the TF CREB5 were enhanced after SCI. Additionally, we confirmed that CREB5 can inhibit neuronal ferroptosis after SCI by promoting the expression of ApoL6, thereby facilitating functional recovery in mice after SCI. In conclusion, our research indicates that targeting CREB5 is a promising approach for the treatment of SCI.

## 2 Materials and Methods

### 2.1 Analysis of scRNA‐Seq and scATAC‐Seq Data

The relevant data were sourced from the Gene Expression Omnibus (GEO) database, specifically datasets GSE234774 and GSE230765 [15]. Following data processing, Uniform Manifold Approximation and Projection (UMAP) plots were employed to visualize the clustering results. Cell type annotations were conducted based on the pre‐uploaded cell annotation information (Meta files) associated with these datasets.

### 2.2 TF Motif Activity Analysis

#### 2.2.1 Data Processing

scATAC‐seq data were processed using Seurat (v4.3) [16] and Signac (v1.6.0) [17]. Neuronal subsets were isolated from the integrated object (scATAC.rds), and chromatin accessibility profiles were visualized via UMAP dimensionality reduction.

#### 2.2.2 Motif Database Integration

Position frequency matrices for vertebrate TFs were retrieved from the JASPAR2020 database (CORE collection) using getMatrixSet, followed by motif annotation to genomic peaks via AddMotifs with the mm10 reference genome (BSgenome. Mmusculus. UCSC. mm10). Motif‐peak associations were stored in the motifs assay slot.

#### 2.2.3 Differential Motif Activity Analysis

For each time point (7d and 2 m post‐injury), differentially accessible peaks (DAPs) were identified against uninjured controls using FindMarkers. Significant DAPs were filtered (adjusted *p*‐value < 0.05, accessibility fraction > 20%) and GC‐content‐corrected by excluding peaks with missing values in GC.percent metadata. Enriched TF motifs were predicted using FindMotifs on significant DAPs, with thresholds of fold‐enrichment > 0.5 and *p*‐value < 0.05. Top‐ranked motifs were selected based on descending fold‐enrichment.

### 2.3 Analysis of TF‐Gene Regulatory Network

First, data preprocessing and integration were performed. The scRNA‐seq counts were processed using Read10X (Seurat), and the TSS‐window ATAC counts were imported via ReadMtx. A Seurat object with dual assays of RNA and ATAC_TSS was constructed, ensuring consistent cell annotations in the metadata. Subsequently, quality control was carried out to filter cells labeled as “Neurons”. For dimensionality reduction and visualization, the RNA modality underwent standardization and scaling, followed by dimensionality reduction using PCA and UMAP. The ATAC modality was processed with TF‐IDF normalization and other procedures, and then reduced in dimensions using LSI and UMAP. In the inference of the transcriptional regulatory network, TFs differentially expressed in neurons were selected. The Spearman correlation between their expression and the accessibility of target gene TSSs was calculated, with only pairs showing a correlation coefficient |r| > 0.3 and a statistical significance of *p* < 0.05 retained to construct a network of significant TF‐gene pairs. The interactions of Creb5 with its top 20 targets were then visualized.

### 2.4 Mice

The 8‐week‐old C57BL/6J wild‐type (WT) mice used in this study were purchased from the Animal Center of Shaoxing People's Hospital. All mice were housed in specific pathogen‐free facilities in the animal center, fed a standard diet, and provided with regular bedding changes. A consistent 12‐h light/dark cycle was maintained with stable temperature and humidity, and animals had free access to food and water. The experimental protocol was approved by the Animal Ethics Committee of Shaoxing People's Hospital and conducted in compliance with the Guide for the Care and Use of Laboratory Animals.

### 2.5 SCI Model

To investigate functional recovery after SCI, severe spinal cord contusion injury was induced at the thoracic level (T10) in isoflurane‐anesthetized mice according to a previously established protocol [18]. Briefly, the skin and musculature were dissected layer‐by‐layer to expose the T10 vertebra and adjacent laminas. Following spinal cord exposure at T10, a controlled impact force of 85 kilodynes (IH‐0400, Precision Systems and Instrumentation) was applied directly to the dorsal surface of the exposed cord. After injury, surgical layers were closed sequentially. Bladder manual expression was performed twice daily until restoration of normal micturition function.

To evaluate pathological recovery after SCI, spinal cord crush injury was induced using a modified protocol based on previously described methods [19]. In brief, after identical surgical exposure of the T10 spinal cord as aforementioned, sustained compression was applied for 2 s using 0.2 mm forceps with calibrated pressure.

### 2.6 Primary Cell

Primary cortical neurons were prepared using modified methods based on previous reports [20]. Under sterile conditions, cerebral cortices were quickly harvested and cut into 0.5–1 mm pieces with sterile scalpels. The tissues were digested at 37°C with 10 μg/mL DNase, and undigested clumps were removed via filtration through meshes. Isolated neurons were seeded onto culture plates at a density of 2 × 10^5^ cells/cm^2^ and cultured in Neurobasal medium containing B‐27 Plus neuronal culture supplement (A3653401, Thermo Fisher Scientific). The cultures were maintained in a 5% CO_2_ incubator, with 50% medium replaced every 48h to sustain metabolic balance and promote neurite extension.

During the isolation of primary spinal cord neurons, we employed the indirect magnetic labeling system (130–115‐389, Miltenyi Biotec) manufactured. Biotinylated microbeads were utilized to magnetically label astrocytes, oligodendrocytes, microglia, endothelial cells, and fibroblasts. Following the labeling procedure, magnetically tagged cells were selectively removed by leveraging the properties of a magnetic field, thereby enabling the acquisition of highly purified, unlabeled primary spinal cord neurons.

### 2.7 Behavioral Assessments

The standardized Basso Mouse Scale (BMS) was used to systematically assess neurological function on days 1, 3, 7, 14, and 28 post‐SCI. The BMS scoring system ranges from 0 to 9 points, where a score of 0 indicates complete loss of hindlimb motor function and paralysis, while a score of 9 represents the restoration of hindlimb function to normal levels. The scoring process was conducted by a trained double‐blind assessment team. Data were obtained by recording and averaging the results of three consecutive dynamic observations, ensuring the objectivity and reliability of the data.

In the rotarod test, an accelerating rotarod apparatus with a variable speed function ranging from 0 to 40 rpm is selected to systematically evaluate the hindlimb motor function and overall motor coordination ability of SCI model mice. Each experimental mouse first undergoes one adaptive practice session, followed by two formal tests with an interval of 20 min. The mean latency of falling from the rod across the two tests quantifies the mice's motor function.

To investigate the impact of SCI on hindlimb tactile sensitivity, we employed the von Frey filament test for assessment. Briefly, each mouse was placed on a metal grid apparatus. Calibrated von Frey filaments were used as the stimulation tools. Each mouse's unilateral hind paw was sequentially subjected to five consecutive stimuli. A positive response was defined as hind paw withdrawal or licking behavior. The von Frey threshold of each mouse was determined as the filament force at which three or more positive responses occurred out of five repeated stimuli.

For hindlimb kinematics, mice walked overground on a 60 × 4 cm runway. Bilateral hindlimb motions were filmed at 60 fps via iPhone. Markers on the crest, hip, knee, ankle, and distal toe tracked movement. Limited hindlimb motion relied on forelimb gait. Videos were analyzed with DeepLabCut for automated landmark tracing, quantifying peak crest/toe height, stride length, and joint angle oscillation per gait cycle. MATLAB generated chronophotographs and stick views of hindlimb movements using selected parameters.

### 2.8 Adenovirus Infection

To knock down CREB5 expression, primary neurons were transfected with adenovirus (GenePharma, China) carrying shRNA‐control (Adv‐shNC) and shRNA‐Creb5 (Adv‐shCREB5). To overexpress ApoL6, adenovirus targeting ApoL6 (Adv‐ApoL6) and a related control (Adv‐Con) were constructed.

### 2.9 Adeno‐Associated Virus Infection

To knockdown (KD) CREB5 expression in neurons in vivo, the adeno‐associated virus (Obio Technology, Shanghai, China) pAAV‐PHP.eB‐hSyn‐shCREB5‐WPRE (AAV‐shCREB5, 9.65 × 10^12^ v.g./mL) was constructed, along with its control pAAV‐PHP.eB‐hSyn‐MCS‐WPRE (AAV‐shNC, 7.44 × 10^12^ v.g./mL). The sequences were shown in Table S1. Similarly, to increase exogenous expression of ApoL6 in vivo, AAV‐PHP.eB‐hSyn‐3xFLAG‐ApoL6‐WPRE (AAV‐ApoL6, 7.32 × 10^12^ v.g./mL) and its control AAV‐PHP.eB‐hSyn‐3xFLAG‐WPRE (AAV‐Con, 6.63 × 10^12^ v.g./mL) were generated. Three days prior to SCI, viral vectors were injected into each horn of the T10 spinal cord segment using a microsyringe pump controller (RWD Life Science, Shenzhen, China) at a rate of 150 nL/min to a depth of 1 mm.

### 2.10 Immunofluorescence Staining

First, anesthetize the experimental mice; then perform perfusion with pre‐cooled normal saline; and subsequently complete the perfusion procedure with 4% paraformaldehyde solution. After perfusion, remove the spinal cord tissue, immerse it in 4% paraformaldehyde solution, and fix it for overnight preservation under low‐temperature conditions. Next, perform dehydration treatment with 15% and 30% sucrose solutions in sequence. After dehydration is completed, embed the sample with optimal cutting temperature compound (Tissue‐Tek; Sakura Finetek, Japan), and then use a slicer to cut it into 15 μm‐thick sections, and rinse the sections with PBS. Place the rinsed sections at room temperature, incubate them in blocking buffer (containing 10% bovine serum albumin and 0.3% Triton‐100) for 1 h, then transfer them to a 4°C environment and incubate them with the primary antibody overnight. The next day, restore the sections to room temperature and incubate them with the secondary antibody for 2 h. Finally, use the THUNDER imaging system (Leica Microsystems GmbH, Wetzlar, Germany) to observe and analyze the spinal cord section samples.

### 2.11 Western Blot

Place the cell samples in ice‐cold RIPA lysis buffer (KeyGEN BioTECH, China). Homogenize the samples thoroughly using an ultrasonic cell disruptor. Subsequently, centrifuge the samples at 4°C for 15 min. Collect the supernatant as the total protein extract. Determine the protein concentration using a BCA protein quantification kit to ensure consistency across all samples. Transfer an appropriate amount of protein samples into new tubes, add 5 × protein loading buffer, mix well, and then denature the proteins by heating the samples in a 95°C metal bath for 10 min. Load equal amounts of the denatured protein samples onto a prepared SDS‐PAGE gel. Run the electrophoresis at 80 V until the samples enter the separating gel, then increase the voltage to 120 V and continue the electrophoresis until the bromophenol blue indicator migrates to the bottom of the gel, thus completing protein separation. Transfer the proteins from the gel to a PVDF membrane using the wet transfer method. After the transfer, block the PVDF membrane in a TBST blocking solution containing 5% skimmed milk on a shaker at room temperature for 2 h. After blocking, immerse the membrane in the diluted primary antibody solution and incubate it on a shaker at 4°C overnight. The next day, wash the membrane three times with TBST buffer for 10 min each. Then, incubate the membrane with horseradish peroxidase (HRP)‐conjugated secondary antibody at room temperature for 1 h. Finally, in a darkroom, use an ECL luminescent reagent (Tanon, China) and a chemiluminescent imaging system for exposure and development.

### 2.12 Reverse Transcription‐Quantitative PCR (qPCR)

Total RNA was extracted from neurons using TRizol reagent (Takara, Japan) according to the manufacturer's protocol. Following purification, the RNA samples were reverse‐transcribed into cDNA using the HiScript II Q RT SuperMix for qPCR kit (Vazyme, China). Real‐time quantitative PCR was performed with the TB Green Premix Ex TaqTM kit. The expression levels of *Creb5* and *ApoL6* mRNA were normalized to the internal control *Actb*, and the relative expression levels of target genes were calculated using the 2^_ΔΔCT^ method.

### 2.13 Detection of ROS and Lipid Peroxides

To assess the ROS levels in primary neurons, a ROS detection kit (S0033S, Beyotime) was employed, and the standardized operating procedures provided by the kit manufacturer were meticulously followed. Flow cytometry was then utilized for detection and analysis, enabling the acquisition of precise quantitative data on ROS. Additionally, a specialized detection kit (ab118970, ab238538, Abcam) was used to measure the levels of lipid peroxidation products in lysates of primary neurons, with strict adherence to the standard experimental protocols outlined by the respective manufacturer.

### 2.14 Axon Microfluid

To investigate axonal growth in neurons, we employed an axonal microfluidic device according to a previously established protocol [21]. Briefly, neurons were seeded on the soma side of the microfluidic device, and neuron culture medium was added as previously described. On the seventh day of cultivation, axons within the axonal compartment were transected using vacuum aspiration. Finally, the regenerative status of the transected axons in the terminal compartment was evaluated via TUJ1 staining.

### 2.15 Live and Dead Staining of Neurons

To achieve visual observation of cell death and quantitative analysis of viable cells, propidium iodide (PI, Beyotime) was employed for staining dead cells, while calcein acetoxymethyl ester (Calcein‐AM, Beyotime) was utilized to label live cells. Following the staining procedure, a fluorescence microscope was used to detect and analyze the stained samples, enabling clear visualization of cell viability status.

### 2.16 Lipolysis Assay

Primary neurons in 12‐well plates were first rinsed with Krebs‐Ringer buffer (KRB: 12 mM HEPES, 121 mM NaCl, 4.9 mM KCl, 1.2 mM MgSO_4_, 0.33 mM CaCl_2_). They were then incubated at 37°C in 300 μL KRB containing 2% fatty acid‐free BSA and 0.1% glucose. The experimental group was treated with 10 μM isoproterenol (Sigma), while the control group received no treatment; both groups were incubated for 1 h. The culture medium was collected to measure FFA and glycerol levels using commercial assay kits (Wako), respectively. For neurons treated with oxygen–glucose deprivation and reperfusion (OGD/R), the same experimental procedures described above were strictly followed.

### 2.17 Seahorse Assay

Referring to established methods [22], a Seahorse analyzer was used to determine FAO rates. Briefly, cells were cultured for 4 h in glucose‐free medium, followed by the addition of 0 mM glucose, 25 mM glucose, or a mixture containing 2‐deoxyglucose (2‐DG, 2 mM) with or without 40 μM etomoxir. After washing, cells were incubated for 30 min at 37°C (without CO₂) in FAO assay medium (111 mM NaCl, 1.25 mM NaH₂PO_4_, 0.5 mM carnitine, 2.5 mM glucose, 5 mM HEPES, pH 7.4) with or without 40 μM etomoxir. Basal mitochondrial respiration was then measured at multiple time points, and antimycin was added to the assay system to determine cellular respiration rates. Finally, the rate of cellular FAO was measured as the oxygen consumption rate using the Seahorse XF24 Extracellular Flux Analyzer.

### 2.18 Quantitative Chromatin Immunoprecipitation (qChIP) Assay

The qChIP assay was performed following the standard protocol (P2078, Beyotime). Neurons were fixed with 1% formaldehyde at room temperature for 10 min, and the reaction was terminated with 1× glycine for 5 min. Whole‐cell lysates were prepared using SDS lysis buffer containing PMSF, and DNA was sheared to 400–800 bp by sonication. After heat‐induced decrosslinking, chromatin was extracted with phenol‐chloroform. The diluted products were incubated with IgG or CREB5 antibody [23] at 4°C with rotation overnight, followed by multiple rounds of washing with low‐salt buffer, high‐salt buffer, lithium salt buffer, and TE buffer sequentially. After elution of the complexes, DNA was purified using the DNA purification kit (D0033, Beyotime), and finally quantitative analysis was performed by qPCR.

### 2.19 Reporter Gene Assay

To study *ApoL6* promoter activity, we constructed WT and mutant promoter reporter vectors and co‐transfected them into HEK293T cells with Lipofectamine3000 (Thermo Fisher). After 6 h of incubation at 37°C in 5% CO_2_, the medium was replaced and cells were cultured for another 48 h. Cells were then washed twice with PBS, lysed with 100 μL of 1 × passive lysis buffer for 15 min, and luciferase activities were measured using the dual‐luciferase reporter assay system (Promega, Madison, WI). The relative fluorescence intensity, calculated as the ratio of firefly to Renilla luciferase activities, was used to analyze *ApoL6* promoter activity and regulatory elements.

### 2.20 ChIP Assay

Treat primary neurons with 1% formaldehyde for protein‐DNA cross‐linking at 37°C for 10 min. The subsequent chromatin immunoprecipitation procedure strictly follows the standardized operating procedures of the Pierce Agarose ChIP Kit (Thermo Fisher). Incubate DNA fragments with the CREB5‐specific antibody or isotype IgG for 12–16 h for specific immunobinding reaction at 4°C. Subsequently, perform qualitative detection of the enriched DNA by PCR. The enrichment level of the target fragment is quantitatively characterized as the relative percentage of the total input DNA.

### 2.21 Statistical Analysis

Data from this study were presented as the mean ± standard deviation (SD) of at least three independent experiments. Statistical analyses were performed using GraphPad Prism 10.0 (GraphPad Software, USA) software. For comparisons between two groups, an unpaired two‐tailed Student's *t*‐test was employed. In the case of multiple groups, one‐way or two‐way analysis of variance (ANOVA) was conducted, followed by Bonferroni post hoc tests. A probability (*p*) value < 0.05 was considered indicative of statistical significance.

## 3 Results

### 3.1 Dimensionality Reduction and Cell Type Annotation of scRNA‐Seq and scATAC‐Seq

**FIGURE 1:**
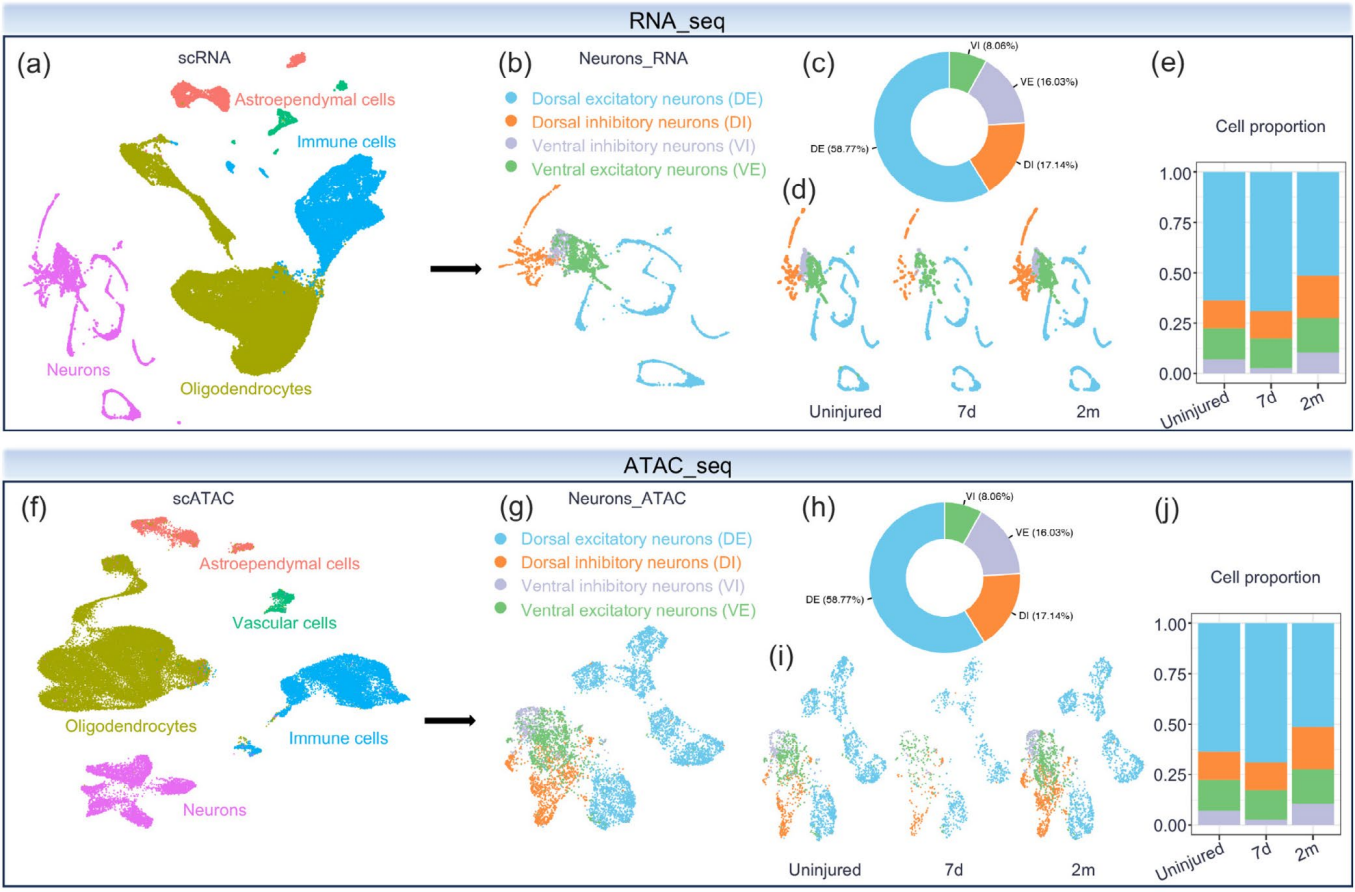
Dimensionality reduction and cell type annotation of scRNA‐seq and scATAC‐seq. (a, b) UMAP plots show all cell types (a) or neurons (b) detected by scRNA‐seq. (c) Pie chart displays the cellular proportions of the four types of neurons. (d) UMAP plot presents the distribution of neurons at the indicated time point. (e) Stacked bar chart shows the cellular proportions of each type of neuron at the indicated time point. (f, g) UMAP plots from scATAC‐seq showing all cells (f) and the four types of neurons (g). (h) Pie chart showing the cellular proportion of each type of neuron. (i) UMAP plots displaying neurons at uninjured, 7 days post‐injury (dpi), and 2 months post‐injury (mpi). (j) Stacked bar chart showing the proportion of neurons at uninjured, 7 dpi, and 2 mpi.

To investigate the gene expression differences in neurons after SCI, we analyzed publicly available scRNA‐seq and scATAC‐seq datasets [15]. For scRNA‐seq data processing, we first performed dimensionality reduction and clustering analysis, followed by cell type annotation using the cell annotations provided by the original study authors (Figure 1a; Figure S1). Subsequently, we isolated the neuronal population for in‐depth analysis, classifying neurons into distinct subtypes and quantifying the proportion of each subtype (Figure 1b–e). Notably, parallel analysis of scATAC‐seq data revealed consistent proportions of different neuronal subtypes, corroborating the findings from scRNA‐seq analysis (Figure 1f–j).

### 3.2 The Expression and Transcriptional Activity of CREB5 in Neurons Are Increased After SCI

**FIGURE 2:**
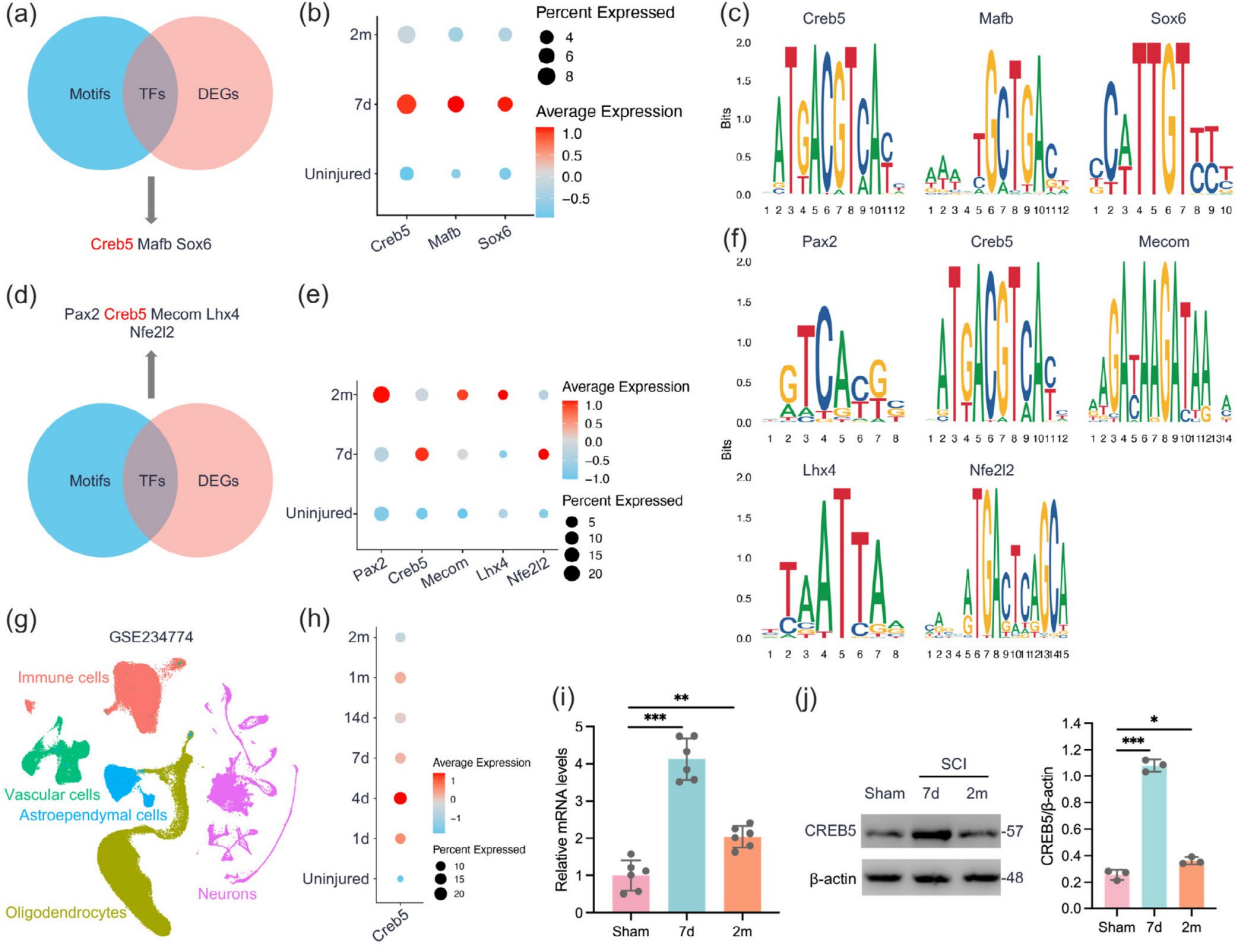
The expression and transcriptional activity of CREB5 in neurons are increased after SCI. (a) Venn plot showing the comparison of differential motifs and DEGs at 7 dpi. (b) Dot plot showing the expression of indicated genes. (c) Motif plot of indicated TFs, sorted in descending order of enrichment fold. (d) Intersection plot of differential motifs and DEGs at 2 mpi. (e) Dot plot of indicated genes. (f) Motif plot showing indicated TFs, sorted in descending order of enrichment fold. (g) UMAP plot of GSE234774 dataset, displaying all cells. (h) Dot plot showing the expression level of *Creb5* in neurons at indicated time points. (i) qPCR results of *Creb5* expression level in neurons (*n* = 6). (j) Western blot results and quantitative statistics of CREB5 in neurons at indicated time points (*n* = 3). Data were analyzed with the one‐way ANOVA (i and j). (*p < 0.05, **p < 0.01, ***p < 0.001).

To identify TF motifs enriched in neuron‐specific DAPs (detected by scATAC‐seq) post‐SCI, we analyzed the overlap between significantly enriched motifs and differentially expressed genes (DEGs) at 7 dpi. The results showed that at 7 dpi, motifs including Creb5, Mafb, and Sox6 were significantly enriched in accessible regions and showed a strong correlation with upregulated DEGs (Figure 2a–c). At 2 mpi, motifs such as Pax2, Creb5, Mecom, Lhx4, and Nfe2l2 were similarly enriched, with their regulatory activity significantly elevated (Figure 2d–f). CREB5 was prioritized for further study due to its consistent enrichment across both acute (7 dpi) and chronic (2 mpi) phases, as well as its known role in neuronal plasticity [24, 25].

Notably, CREB5 expression post‐SCI exhibits a biphasic trend (initial upregulation followed by downregulation; Figure 2b–e), which is validated in both the GSE234774 dataset and qPCR experiments (Figure 2g–i). Furthermore, Western blot analysis confirmed that the expression level of CREB5 indeed increased after SCI and then decreased at 2 mpi, which is consistent with the expression trend of *CREB5* mRNA (Figure 2j). In conclusion, the expression of the TF CREB5 increases after SCI, and its transcriptional activity is enhanced.

### 3.3 KD Of CREB5 in Vitro Impairs Axonal Growth and Exacerbates Neuronal Death

To investigate the impact of CREB5 on axonal growth and neuronal survival after injury, we knocked down CREB5 in primary neurons treated with OGD/R in vitro and established an axon microfluid experimental model (Figures S2a–c). The results demonstrated that CREB5 KD significantly inhibited the axonal growth of neurons (Figure S2d). Furthermore, we also found that CREB5 KD exacerbated neuronal death (Figure S2e). In further studies, we performed CREB5 overexpression in neurons and found that it significantly promoted axonal growth and alleviated neuronal death (Figures S2f–g).

### 3.4 CREB5 KD Impeded Functional Recovery of Mice After SCI

**FIGURE 3:**
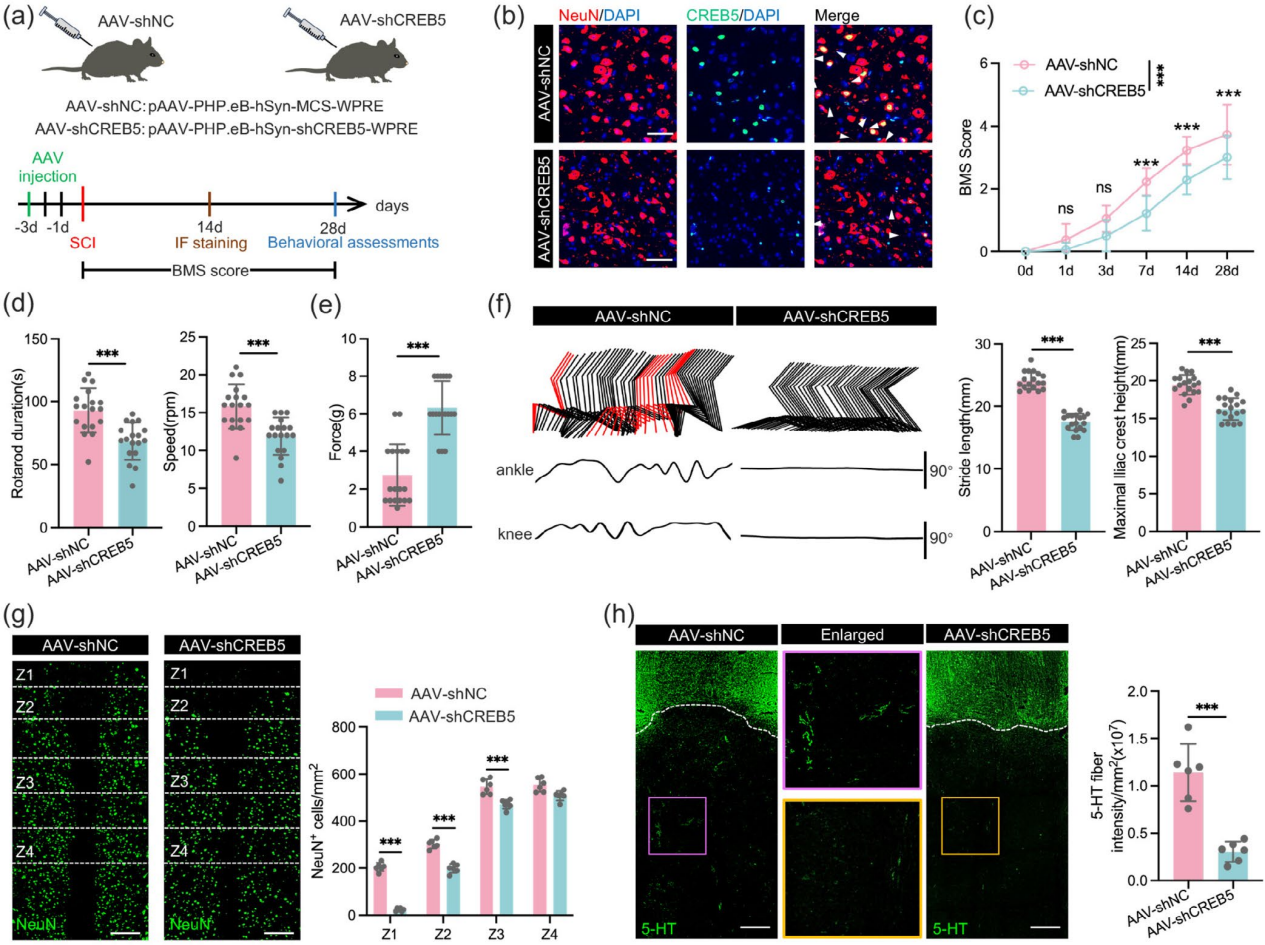
CREB5 KD impeded functional recovery of mice after SCI. (a) Schematic diagram of experimental grouping and procedures. (b) Immunofluorescence results showing the in vivo KD efficiency of CREB5 in neurons. Scale bar = 50 μm. (c) BMS score results of the CREB5 KD group and negative control (NC) group in neurons at indicated time points (*n* = 18). (d) Duration and final speed results of the rotarod test in each group of mice at 28 dpi (*n* = 18). (e) Bar chart of von Frey test results of the two groups of mice at 28 dpi (*n* = 18). (f) For mice 28 days after injury, the analysis related to hindlimb movement includes the decomposition and presentation of color‐coded stick diagrams of hindlimb movement, the recording of joint angle oscillation trajectories of the ankle and knee joints, and the quantitative statistics of stride length and maximum iliac crest height (*n* = 18). (g) At 14 dpi, spinal cord neurons of mice in the AAV‐shNC group and AAV‐shCREB5 group were subjected to immunofluorescence staining with indicated antibody, and the number of surviving neurons in the Z1‐Z4 zone near the injury was quantitatively analyzed (*n* = 6). Scale bar = 200 μm. (h) Representative images of the distal injury area labeled with anti‐5‐hydroxytryptamine (5‐HT) antibody at 28 dpi (*n* = 6). Scale bar = 200 μm. Data were analyzed using the two‐tailed unpaired Student's *t*‐test (d, e, f, and h) and two‐way ANOVA (c and g) followed by post hoc Bonferroni correction. (**p < 0.01, ***p < 0.001).

To investigate the effect of CREB5 on functional recovery in mice after SCI, we constructed an adeno‐associated virus (AAV) targeting the knockdown of neuronal CREB5 (Figure 3a,b). Studies have confirmed that CREB5 KD hinders the recovery of hindlimb function in mice, and this conclusion is validated by BMS scores, rotarod tests (Figure 3c,d). In addition, KD of neuronal CREB5 also impedes the recovery of sensory function after SCI (Figure 3e). Notably, at 28 dpi, hindlimb kinematics analysis revealed multiple motor function impairments in mice with neuronal CREB5 KD. Specifically, they exhibited reduced ankle joint range of motion, shorter stride length, lower maximum iliac crest height, and decreased hindlimb joint swing amplitude (Figure 3f).

To verify the above speculation, we evaluated the survival of neurons in specific zones (Z1‐Z4) through immunofluorescence staining according to the method described previously [26]. The results confirmed that CREB5 KD is detrimental to neuronal survival (Figure 3g). Further assays showed that KD of neuronal CREB5 has an adverse effect on the regeneration of 5‐HT^+^ serotonergic axons (Figure 3h). In conclusion, our study demonstrates that neuronal CREB5 KD impairs the recovery of hindlimb function in mice after SCI.

### 3.5 CREB5 Promotes ApoL6 Expression by Transactivating *ApoL6* Transcription After SCI

**FIGURE 4:**
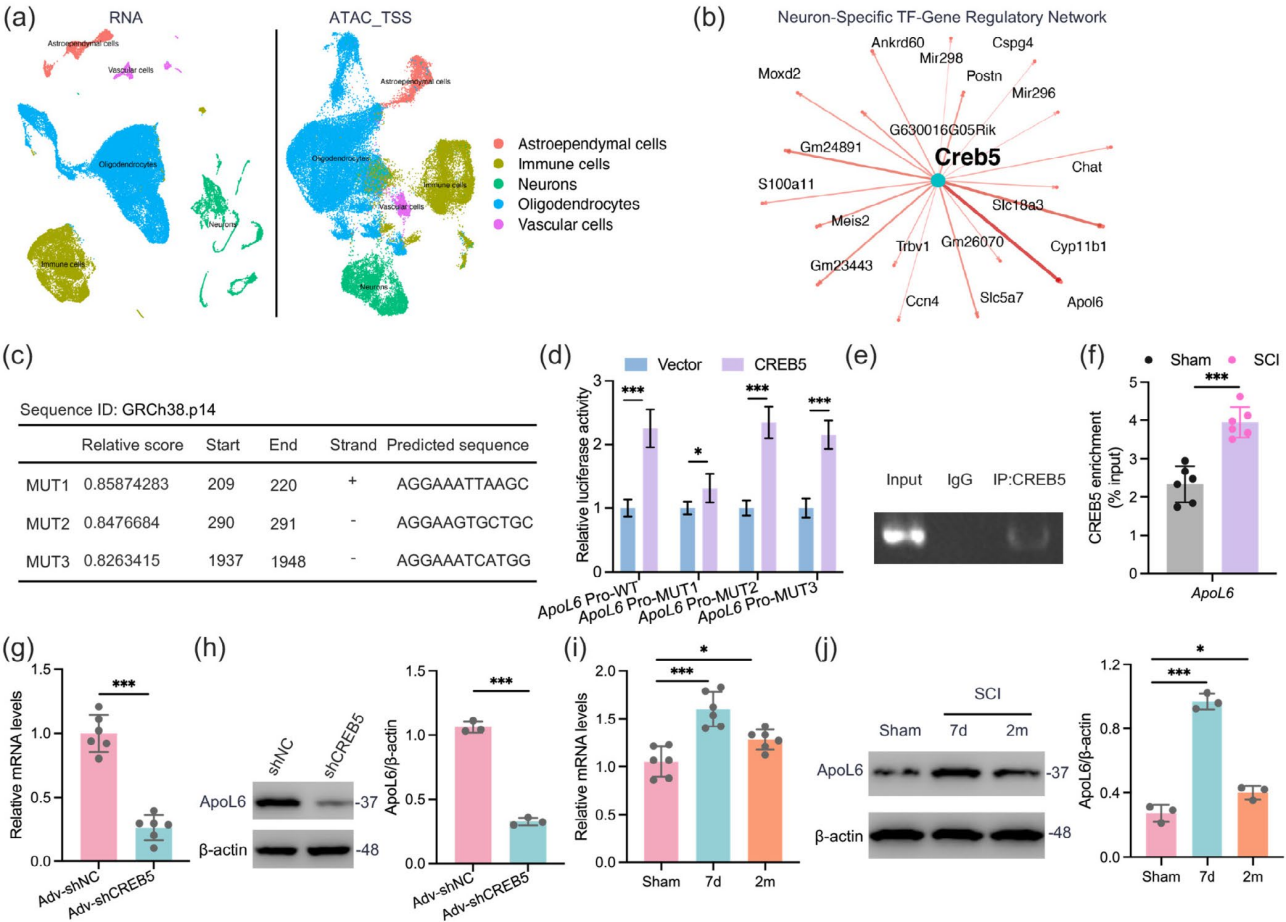
CREB5 promotes ApoL6 expression by transactivating Apol6 transcription after SCI. (a) UMAP plot showing the integrated analysis results of scRNA and TSS window of scATAC. (b) Network diagram of *Creb5* regulating downstream genes. (c) Table of potential CREB5 binding sites in the *ApoL6* promoter region. (d) After transfecting WT *ApoL6*‐Pro or mutant *ApoL6*‐Pro plasmids into HEK293T cells, the expression level of the reporter gene was determined by dual‐luciferase assay (*n* = 6). (e) ChIP assay verified the binding of CREB5 to the *Apol6* promoter. (f) Neurons were isolated from the spinal cord tissues of the sham group and 7 dpi group by magnetic bead system, and qChIP analysis was performed on *Apol6* (*n* = 6). (g) qPCR results of *Apol6* expression levels in neurons isolated by magnetic bead system at 7 dpi with or without CREB5 KD (*n* = 6). (h) Western blot results and quantitative statistics of ApoL6 in neurons at 7 dpi (*n* = 3). (i) qPCR results of *Apol6* in neurons at indicated injury time points (*n* = 6). (j) Western blot results and quantitative statistics of neurons at indicated injury time points (*n* = 3). Data were analyzed using the two‐tailed unpaired Student's *t*‐test (f, g, and h), as well as one‐way ANOVA (i and j) and two‐way ANOVA (d) followed by post hoc Bonferroni correction. (*p < 0.05, ***p < 0.001).

Our study revealed that the expression and transcriptional activity of CREB5 are upregulated following SCI, and CREB5's impact on functional recovery in SCI mice has been validated. To elucidate the regulatory mechanisms by which CREB5 modulates neuronal survival and functional recovery in mice after SCI, we integrated scRNA‐seq and scATAC_TSS window data to construct a gene co‐expression network (Figure 4a,b). The results identified *ApoL6* as a potential transcriptional target regulated by CREB5 (Figure 4b). Moreover, we constructed *ApoL6* promoter mutants based on binding site predictions from the JASPAR database (Figure 4c). Luciferase reporter assays showed that CREB5 overexpression significantly enhanced the activity of the WT *ApoL6* promoter, whereas this activation effect was not significantly different from the control group in the Pro‐MUT1 mutant (Figure 4d). ChIP assays confirmed the binding interaction between CREB5 and the promoter region of *ApoL6* in neurons (Figure 4e). Notably, the results of qChIP assays showed that the binding level of CREB5 to the *ApoL6* promoter in post‐injury neurons was significantly higher than that in uninjured neurons (Figure 4f). Further investigation demonstrated that CREB5 KD in neurons significantly decreased the mRNA and protein levels of *ApoL6* (Figure 4g,h). Notably, CREB5 levels increased after SCI and decreased at 2 mpi, a trend that aligned with the changes in *ApoL6* mRNA and protein levels (Figure 4i,j). Collectively, our findings indicate that after SCI, CREB5 enhances the transcription of *ApoL6*, ultimately upregulating ApoL6 expression.

### 3.6 CREB5 Inhibits Neuronal Ferroptosis by Promoting ApoL6 Expression After SCI

**FIGURE 5:**
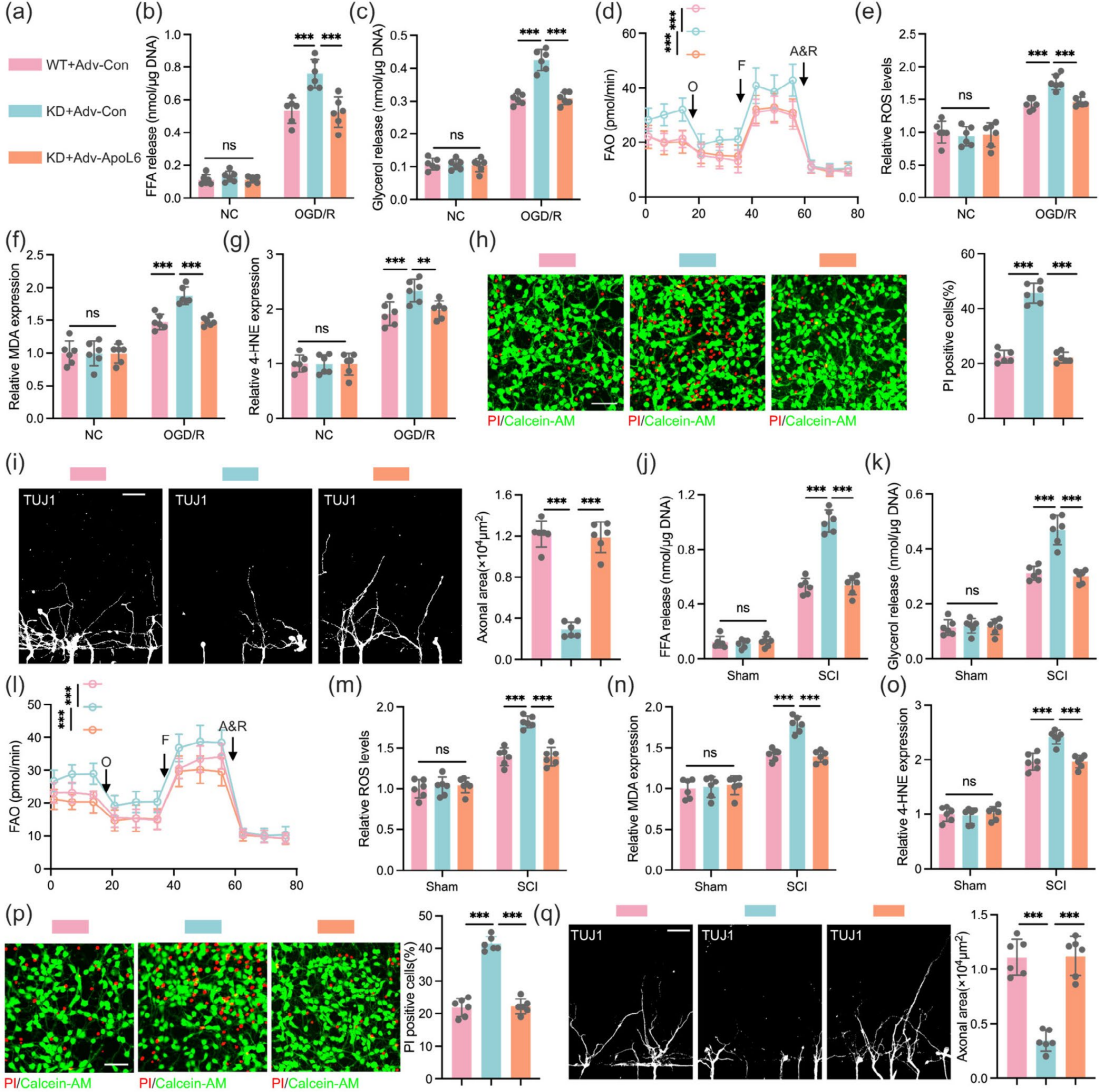
CREB5 inhibits neuronal ferroptosis by promoting ApoL6 expression after SCI. (a) Schematic diagram of experimental groups, with different colors representing indicated experimental groups. (b, c) Detection results of FFA and glycerol release in neurons after OGD/R treatment or control treatment, respectively (*n* = 6). (d) Detection results of neuronal FAO levels in experimental groups with different genotypes (*n* = 6). (e‐g) Bar graphs respectively show the detection results of ROS, MDA and 4‐HNE contents in neurons after OGD/R treatment or NC treatment (*n* = 6). (h) PI/Calcein‐AM double staining shows the death ratio of neurons with different genotypes after OGD/R treatment and the quantitative statistics chart (*n* = 6). Scale bar = 50 μm. (i) After OGD/R treatment of neurons, the axonal growth of different genotypes in the microfluidic device was observed by anti‐TUJ1 antibody labeling (*n* = 6). Scale bar = 100 μm. (j‐q) For mice injected with AAV‐shCREB5, spinal cord tissues from the sham group or those at 7 days after SCI were collected. Neurons were isolated using a magnetic bead system and infected with different Adv respectively for the following experimental evaluations. (j, k) Detection results of FFA and glycerol release contents in neurons (*n* = 6). (l) FAO levels in neurons of different genotypes after injury (*n* = 6). (m‐o) Detection results of ROS, MDA and 4‐HNE contents (*n* = 6). (p) Death ratios of neurons with different genotypes after injury (*n* = 6). Scale bar = 50 μm. (q) Comparison of axonal growth of neurons with different genotypes in microfluidic devices after injury (*n* = 6). Scale bar = 100 μm. Data were analyzed using one‐way ANOVA (b, c, e‐k, m‐q) and two‐way ANOVA (d and l) followed by post hoc Bonferroni correction. (**p < 0.01, ***p < 0.001).

To determine whether the effect of CREB5 on neuronal death and axonal regeneration is achieved by regulating the expression of ApoL6, we constructed primary neurons with CREB5 KD and ApoL6 overexpression via adenovirus infection, and the indicated experimental groups are shown in Figure 5a. Notably, we first confirmed that CREB5 KD indeed reduced LD levels in neurons following SCI (Figures S3a–b). Furthermore, following the previously established experimental protocol, we injected the non‐selective β‐adrenergic agonist isoproterenol to artificially stimulate lipolysis [9, 27]. After administering isoproterenol stimulation, we found that CREB5 KD significantly increased FFA and glycerol content, while ApoL6 replenishment reduced FFA and glycerol release (Figures S4a–b). Results from the seahorse assays confirmed that CREB5 KD enhanced FAO in neurons, and this effect was reversed by ApoL6 supplementation (Figure S4c). It is known that neurons are highly sensitive to FAO, and excessively high FAO leads to elevated ROS levels and lipid peroxidation [12, 13], which were also verified by our ROS detection and lipid peroxide detection experiments (Figures S4d–f). Furthermore, results from Calcein‐AM/PI double staining and axon microfluid assays showed that CREB5 KD exacerbated neuronal death and axonal growth impairment, while overexpression of ApoL6 could eliminate these adverse effects (Figures S4g–i).

In further studies, we used the OGD/R model to simulate neuronal injury after SCI. Consistent with the previous experimental results, the release of FFA and glycerol from neurons significantly increased after CREB5 KD, while ApoL6 supplementation could inhibit this increase in FFA and glycerol release (Figure 5b,c). The seahorse assays also confirmed that CREB5 KD upregulated FAO in neurons, and ApoL6 replenishment eliminated the effect of CREB5 KD (Figure 5d). In addition, the detection of neuronal ferroptosis indicators showed that CREB5 KD exacerbated neuronal ferroptosis and axonal growth impairment, whereas overexpression of ApoL6 could alleviate the adverse effects caused by neuronal injury after CREB5 KD (Figure 5e–i). We isolated primary neurons after SCI using a magnetic labeling system. The results were consistent with the above findings that CREB5 KD after SCI promoted the release of FFA and glycerol from LDs, increased FAO in neurons, and exacerbated neuronal ferroptosis and axonal growth impairment (Figure 5j–q). Notably, treatment with specific ferroptosis inhibitors, Liproxstatin‐1 or Ferrostatin‐1, suppresses neuronal ferroptosis and prevents the damage caused by CREB5 KD (Figures S4j–m). In summary, our study confirms that CREB5 can inhibit neuronal ferroptosis by promoting the expression of ApoL6 after SCI.

### 3.7 CREB5 Promotes Functional Recovery in Mice After SCI by Enhancing the Expression of ApoL6

**FIGURE 6:**
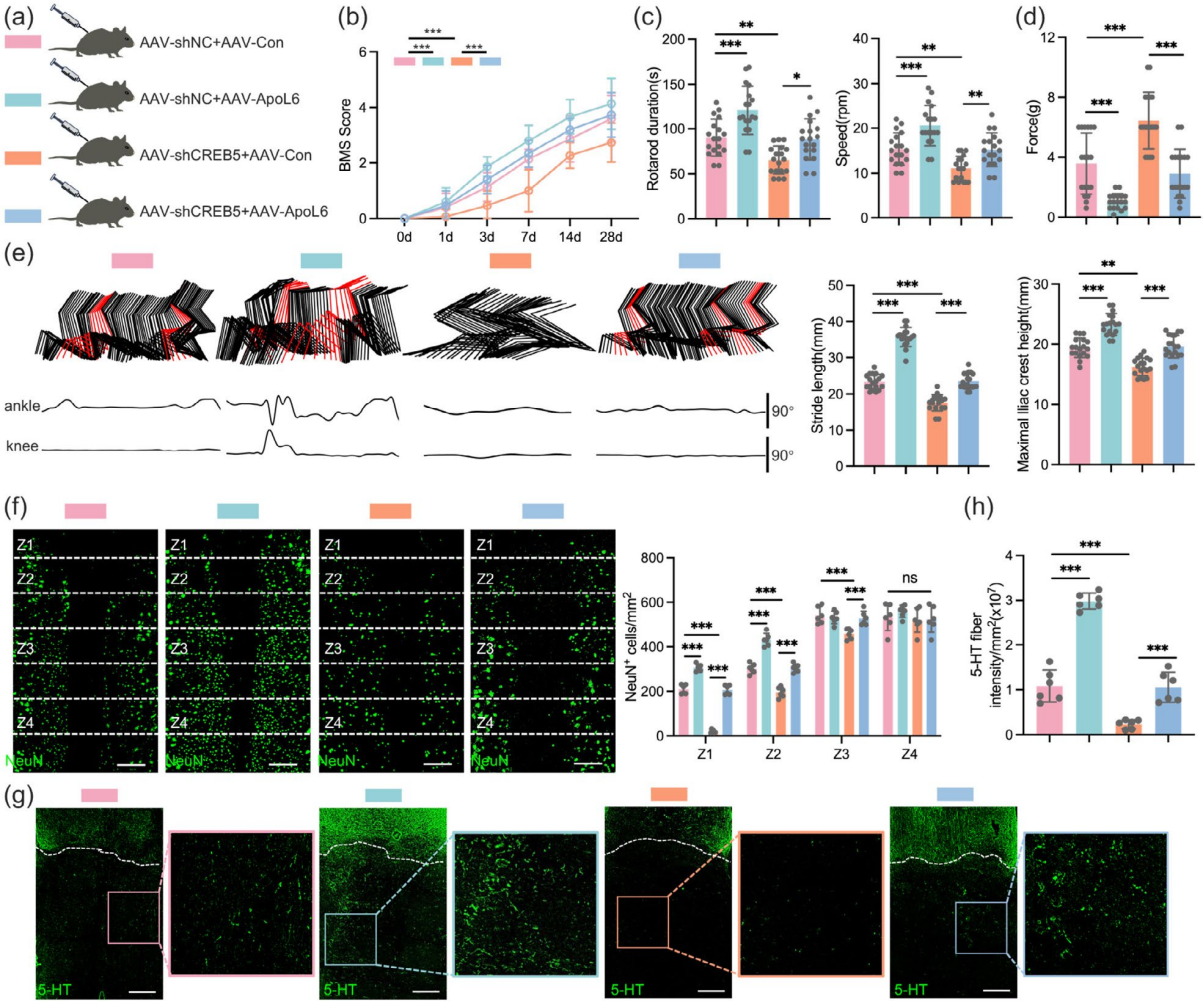
CREB5 promotes functional recovery in mice after SCI by enhancing the expression of ApoL6. (a) Schematic diagram of experimental groups. (b) Comparison of BMS scores among different experimental groups at indicated time points after injury (*n* = 18). (c) Rotarod test was performed on mice at 28 dpi (*n* = 18). (d) Results of von Fery test for each group of mice at 28 dpi (*n* = 18). (e) In mice 28 days post‐injury, the evaluation concerning hindlimb motion comprises the breakdown and display of color‐coded stick graphs depicting hindlimb movement, the documentation of joint angle oscillation paths of the ankle and knee joints, as well as the quantitative data of stride length and maximum iliac crest height (*n* = 18). (f) At 14 days after injury, neurons in spinal cord tissues were labeled with anti‐NeuN antibody, and the number of neurons in the indicated area near the injury area was counted (*n* = 6). Scale bar = 200 μm. (g, h) At 28 days after injury, 5‐HT^+^ serotonergic axons in the distal part of the injury lesion were labeled with anti‐5‐HT antibody (*n* = 6). Scale bar = 200 μm. Data were analyzed using one‐way ANOVA (c, d, e, f, and h) and two‐way ANOVA (b) followed by post hoc Bonferroni correction. (*p < 0.05, **p < 0.01, ***p < 0.001).

To investigate whether the effect of CREB5 on functional recovery in mice after SCI is achieved by promoting the expression of ApoL6, we constructed adeno‐associated viruses to obtain in vivo CREB5 KD mice and corresponding control mice, followed by injection of AAV‐Con or AAV‐ApoL6 (Figure 6a; Figures S5a–b). A series of functional experiments showed that CREB5 KD significantly hindered the recovery of hindlimb function in mice, while overexpression of ApoL6 not only partially promoted hindlimb functional recovery in mice after SCI but also partially mitigated the adverse effects caused by CREB5 KD (Figure 6b–d). Furthermore, CREB5 KD reduces hindlimb swing, narrows the range of motion of the ankle and knee joints, and decreases stride length and maximum iliac crest height in mice. However, ectopic overexpression of ApoL6 after injury enables mice to exhibit walking movements, occasional plantar stepping, an expanded range of ankle motion, increased stride length, and improved body support, while also offsetting the adverse effects caused by CREB5 KD (Figure 6e).

Notably, immunofluorescence results confirmed that CREB5 KD increased neuronal death after SCI and hindered the regeneration of 5‐HT^+^ serotonergic axons, whereas ectopic expression of ApoL6 promoted neuronal survival and axonal regeneration (Figure 6f–h). In summary, our study found that after SCI, CREB5 reduces neuronal death and promotes 5‐HT^+^ serotonergic axonal regeneration by promoting the expression of ApoL6.

## 4 Discussion

In this study, we identified CREB5 as a key target for inhibiting neuronal ferroptosis after SCI. Specifically, after SCI, CREB5 upregulates the expression of ApoL6 by enhancing its transcriptional activity on *ApoL6*. ApoL6, a key inhibitor of LD catabolism, potently inhibits excessive ROS production and lipid peroxidation in neurons post‐SCI, thereby reducing neuronal sensitivity to ferroptosis. In summary, the CREB5/ApoL6 axis does not broadly suppress ferroptosis. Instead, it precisely regulates neuronal LD metabolism to maintain energy metabolic homeostasis, establishing a specific regulatory cascade (“lipid metabolic balance → ferroptosis suppression”). This cascade promotes neuronal survival and axonal regeneration, ultimately facilitating functional recovery. Therefore, we consider the CREB5/ApoL6 axis a target worthy of in‐depth research in the treatment of SCI.

Ferroptosis is a novel form of cell death driven by iron‐dependent lipid peroxidation. As organelles for neutral lipid storage, LDs participate in the process of ferroptosis by regulating the metabolism of polyunsaturated fatty acids (PUFAs) and free radicals. Studies have shown that LDs can reduce the possibility of free PUFAs (such as arachidonic acid) participating in lipid peroxidation through sequestration, thereby inhibiting ferroptosis [28]. Conversely, PUFAs released by LD hydrolysis can be esterified into membrane phospholipids, which form lipid peroxides under the catalysis of ACSL4, thus promoting ferroptosis [29]. In addition, the LD surface protein PLIN2 maintains cellular antioxidant capacity by inhibiting lipolysis, and its knockout can enhance ferroptosis sensitivity [28]. Furthermore, lipid transfer between LDs and mitochondria finely regulates the ferroptosis threshold by modulating the supply of lipid peroxidation substrates [30].

LDs are a type of dynamic organelle that are widely found in most eukaryotic and prokaryotic cells. In neurons and glial cells, LDs serve as the main storage site for neutral lipids and play an important role in cellular metabolism [13, 31]. LDs can not only act as energy reservoirs but also sequester toxic fatty acids by esterifying FFA, ultimately preventing the occurrence of intracellular lipid peroxidation [32]. During oxidative stress in neurons after SCI, lipid accumulation occurs, and the formation of LDs can effectively prevent lipid peroxidation in neurons [33]. Furthermore, studies have reported that mutations in two key lipolytic genes, *atgl‐1* and *lid‐1*, lead to restricted lipolysis of LDs, which protects neurons from neurodegeneration caused by overactivation [34]. In one study, it was found that after brain injury, LDs accumulate in neurons, which increases β‐oxidation and oxidative phosphorylation. However, excessive oxidative phosphorylation increases the production of ROS, thereby leading to enhanced lipid peroxidation and cell apoptosis [35]. After SCI, the blood‐spinal cord barrier is disrupted, impairing the glucose supply to neurons [10, 36]. Under such circumstances, neurons can obtain energy from alternative energy substrates. Previous studies have confirmed that neurons can acquire lactate as an energy substrate through the glial cell‐lactate shuttle system [19, 37, 38]. Although there are reports indicating that neurons can obtain energy via lipid metabolism, the production of FFA through lipolysis of LDs induces lipid peroxidation and increased ROS, which further exacerbates neuronal death [34, 35]. To prevent severe oxidative stress in neurons, lipids are usually stored in LDs or transported into glial cells [39]. Moreover, evidence has shown that neurons transfer lipids to neuroglial cells to avoid excessive lipid accumulation in themselves [40]. ApoL6, a member of the apolipoprotein L (ApoL) family, has been proven to strongly inhibit the lipolysis of LDs [9]. Our study demonstrates that elevated ApoL6 expression post‐SCI reduces excessive FAO by inhibiting aberrant FFA and glycerol release in neurons, consequently decreasing ROS production. In addition, it can inhibit the release of toxic fatty acids, effectively alleviate lipid peroxidation, and ultimately promote functional recovery by alleviating neuronal ferroptosis. Therefore, we propose that the regulation of LD catabolism and anabolism after SCI may represent a self‐protective mechanism of neurons, which deserves further in‐depth research.

The main function of the transcription factor CREB5 is to regulate gene transcription by binding to specific DNA sequences. It can recognize and bind to cAMP response elements, thereby affecting the expression of downstream genes—a process often closely associated with cellular stress responses, metabolic regulation, and growth and development. Unlike other members of the CREB family, CREB5 lacks a conserved PKA‐regulated phosphorylation domain and thus cannot respond to the activation of the cAMP‐PKA signaling pathway [23, 41, 42]. In the neural field, studies have confirmed its role in regulating neural plasticity during mouse embryonic development, and its dysregulation may be associated with carcinogenesis [25, 42, 43]. Notably, CREB5 also plays an important role in the metabolic field. Studies have reported that semaglutide enhances myocardial metabolism by targeting *Creb5*/NR4a1, providing a therapeutic approach for heart failure [44]. Additionally, a study found that fatty acid β‐oxidation in diabetic patients may be closely related to CREB5 [45]. Interestingly, research in the food field has also confirmed that CREB5 promotes fat deposition in cells [46]. Our study revealed that CREB5 expression is upregulated after SCI, and KD of CREB5 increases neuronal ferroptosis, restricting regenerative repair and functional recovery in mice after injury. Further studies demonstrated that after SCI, CREB5 inhibits neuronal lipid peroxidation and excessive ROS production by enhancing transcriptional activity on the lipolysis‐related protein ApoL6 and regulates the metabolic balance of neuronal LDs. This provides a new perspective for research on inhibiting neuronal ferroptosis after SCI. However, our study has limitations. The specific mechanisms regulating CREB5 expression post‐SCI remain unclear. For instance, miR‐206 has been shown to downregulate CREB5 and inhibit PI3K/AKT signaling pathway activation in hepatocellular carcinoma [47]. In addition, DNA methylation is also involved in the regulation of CREB5 [48]. It is worth noting that histone modifications have also been reported to regulate CREB5 expression [24]. Therefore, the regulation of CREB5 expression after SCI may involve multiple factors, and we believe this direction deserves in‐depth research.

In conclusion, this study emphasizes that after SCI, both the transcriptional activity and expression level of the neuronal transcription factor CREB5 are increased, which effectively upregulates the expression of ApoL6, thereby restricting LD decomposition, reducing the production of ROS and lipid peroxidation, and ultimately inhibiting neuronal ferroptosis. Therefore, CREB5 is expected to be a new potential target for inhibiting ferroptosis after SCI.

## Author Contributions

Conceptualization, X.X. and X.S.; methodology, X.X. and Z.C.; investigation, X.X., X.G., Z.C., F.W. and X.S.; visualization, Z.C. and C.W.; funding acquisition, X.S.; project administration, X.S.; writing‐original draft, X.X., Z.C. and C.W.; writing‐review editing, X.S.; supervision, X.S. All authors approved the final version of the manuscript.

## Funding

This work was supported by Medical Science and Technology Project of Zhejiang Province (Grant Nos. 2023KY349 and 2023KY359).

## Consent

The study was approved by the Institutional Animal Care and Use Committee of Shaoxing People's Hospital and conducted in accordance with the Guide for the Care and Use of Laboratory Animals (Ethics Approval Number: 2024Z055).

## Conflicts of Interest

The authors declare no conflicts of interest.

## Supporting information


**Figure S1:** Single‐cell RNA sequencing cell type markers. Dot plot showing cell annotation markers in Figure 1a.
**Figure S2:** CREB5 is beneficial to the survival of neurons and axonal growth in vitro. (a) β‐tubulin III validates neuronal identity. Scale bar = 50 μm. (b) After knocking down CREB5 in neurons in vitro, the knockdown efficiency was verified by Western blot (*n* = 3). (c) Schematic diagram of the microfluidic device. (d) Axonal growth of neurons in the CREB5 knockdown group and control group, detected by anti‐TUJ1 antibody labeling (*n* = 6). Scale bar = 100 μm. (e) Death ratios of neurons in the CREB5 knockdown group and control group, shown by PI/Calcein double staining (*n* = 6). Scale bar = 50 μm. (f) Axonal growth of neurons in the CREB5 overexpression group and control group, detected by anti‐TUJ1 antibody labeling (*n* = 6). Scale bar = 100 μm. (g) Death ratios of neurons in the CREB5 overexpression group and control group, shown by PI/Calcein double staining (*n* = 6). Scale bar = 50 μm. Data were analyzed using the two‐tailed unpaired Student's *t*‐test (b, d, e, f and g). (***p < 0.001).
**Figure S3:** Knockdown of CREB5 promotes lipid droplet breakdown. (a) Use BODIPY to detect lipid droplet levels in primary neurons from the control group and the CREB5 knockdown group. Scale bar = 5 μm (*n* = 6). (b) BODIPY and anti‐MAP2 antibody were used to detect LD levels in neurons located 2 mm from the epicenter of SCI. Scale bar = 20 μm. Data were analyzed using the two‐tailed unpaired Student's *t*‐test (a). (***p < 0.001).
**Figure S4:** CREB5 inhibits neuronal ferroptosis by promoting ApoL6 expression. (a, b) Detection results of FFA and glycerol release in neurons after isoproterenol treatment or control treatment, respectively (*n* = 6). (c) Detection results of neuronal FAO levels in experimental groups with different genotypes (*n* = 6). (d‐f) Bar graphs respectively show the detection results of ROS, MDA and 4‐HNE contents in neurons after isoproterenol treatment or NC treatment (*n* = 6). (g, h) PI/Calcein‐AM double staining shows the death ratio of neurons with different genotypes after isoproterenol treatment and the quantitative statistics chart (*n* = 6). (i) After isoproterenol treatment of neurons, the axonal growth of different genotypes in the microfluidic device was observed by anti‐TUJ1 antibody labeling (*n* = 6). (j) Schematic diagram of the grouping. (k‐m) Bar chart showing the levels of ROS, MDA, and 4‐HNE in neurons. Data were analyzed using one‐way ANOVA (a, b, d, e, f, h, i, k, l and m) and two‐way ANOVA (c) followed by post hoc Bonferroni correction. (*p < 0.05, **p < 0.01, ***p < 0.001).
**Figure S5:** Overexpression of ApoL6 in neurons. (a) Confocal images show the efficiency of ApoL6 overexpression in neurons (*n* = 6). Scale bar = 5 μm. (b) The transfection efficiency of the control group and the overexpressing ApoL6 group was detected by anti‐Flag and anti‐NeuN antibody labeling. Scale bar = 50 μm. Data were analyzed using the two‐tailed unpaired Student's *t*‐test (a). (***p < 0.001).
**Table S1:** Nucleotide sequences of shRNA.

## Data Availability

The data that support the findings of this study are available from the corresponding author upon reasonable request.
