# Supplementary material for: CREB5 Inhibits Neuronal Ferroptosis via Transactivating ApoL6 to Regulate Lipid Droplet Metabolism After Spinal Cord Injury
Source: CNS Neurosci Ther. 2026 Feb 17;32(2):e70783. doi: 10.1002/cns.70783 (PMC12910403; doi:10.1002/cns.70783)
Supplement: Supplementary file 1 — Figure S1: Single‐cell RNA sequencing cell type markers. Dot plot showing cell annotation markers in Figure 1a. Figure S2: CREB5 is beneficial to the survival of neurons and axonal growth in vitro. (a) β‐tubulin III validates neuronal identity. Scale bar = 50 μm. (b) After knocking down CREB5 in neurons in vitro, the knockdown efficiency was verified by Western blot (n = 3). (c) Schematic diagram of the microfluidic device. (d) Axonal growth of neurons in the CREB5 knockdown group and control group, detected by anti‐TUJ1 antibody labeling (n = 6). Scale bar = 100 μm. (e) Death ratios of neurons in the CREB5 knockdown group and control group, shown by PI/Calcein double staining (n = 6). Scale bar = 50 μm. (f) Axonal growth of neurons in the CREB5 overexpression group and control group, detected by anti‐TUJ1 antibody labeling (n = 6). Scale bar = 100 μm. (g) Death ratios of neurons in the CREB5 overexpression group and control group, shown by PI/Calcein double staining (n = 6). Scale bar = 50 μm. Data were analyzed using the two‐tailed unpaired Student's t‐test (b, d, e, f and g). (***p < 0.001). Figure S3: Knockdown of CREB5 promotes lipid droplet breakdown. (a) Use BODIPY to detect lipid droplet levels in primary neurons from the control group and the CREB5 knockdown group. Scale bar = 5 μm (n = 6). (b) BODIPY and anti‐MAP2 antibody were used to detect LD levels in neurons located 2 mm from the epicenter of SCI. Scale bar = 20 μm. Data were analyzed using the two‐tailed unpaired Student's t‐test (a). (***p < 0.001). Figure S4: CREB5 inhibits neuronal ferroptosis by promoting ApoL6 expression. (a, b) Detection results of FFA and glycerol release in neurons after isoproterenol treatment or control treatment, respectively (n = 6). (c) Detection results of neuronal FAO levels in experimental groups with different genotypes (n = 6). (d‐f) Bar graphs respectively show the detection results of ROS, MDA and 4‐HNE contents in neurons after isoproterenol treatment or NC treat [file CNS-32-e70783-s001.docx]

**
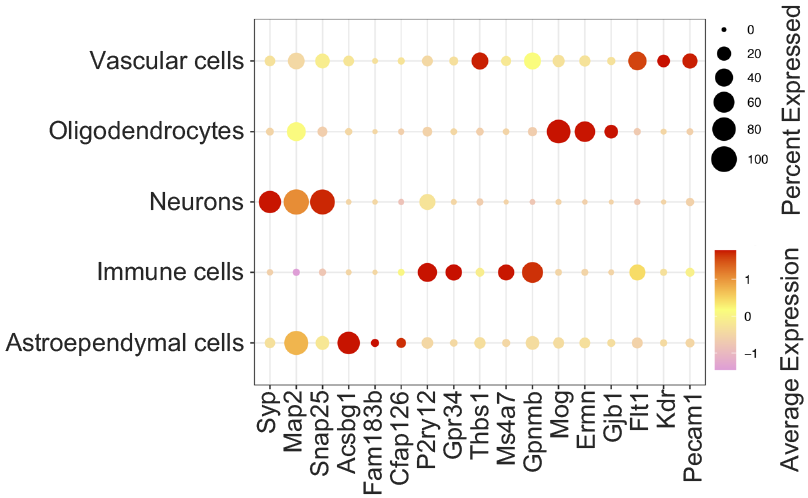
Fig. S1 Single-cell RNA sequencing cell type markers.** Dot plot showing cell annotation markers in Figure 1a.


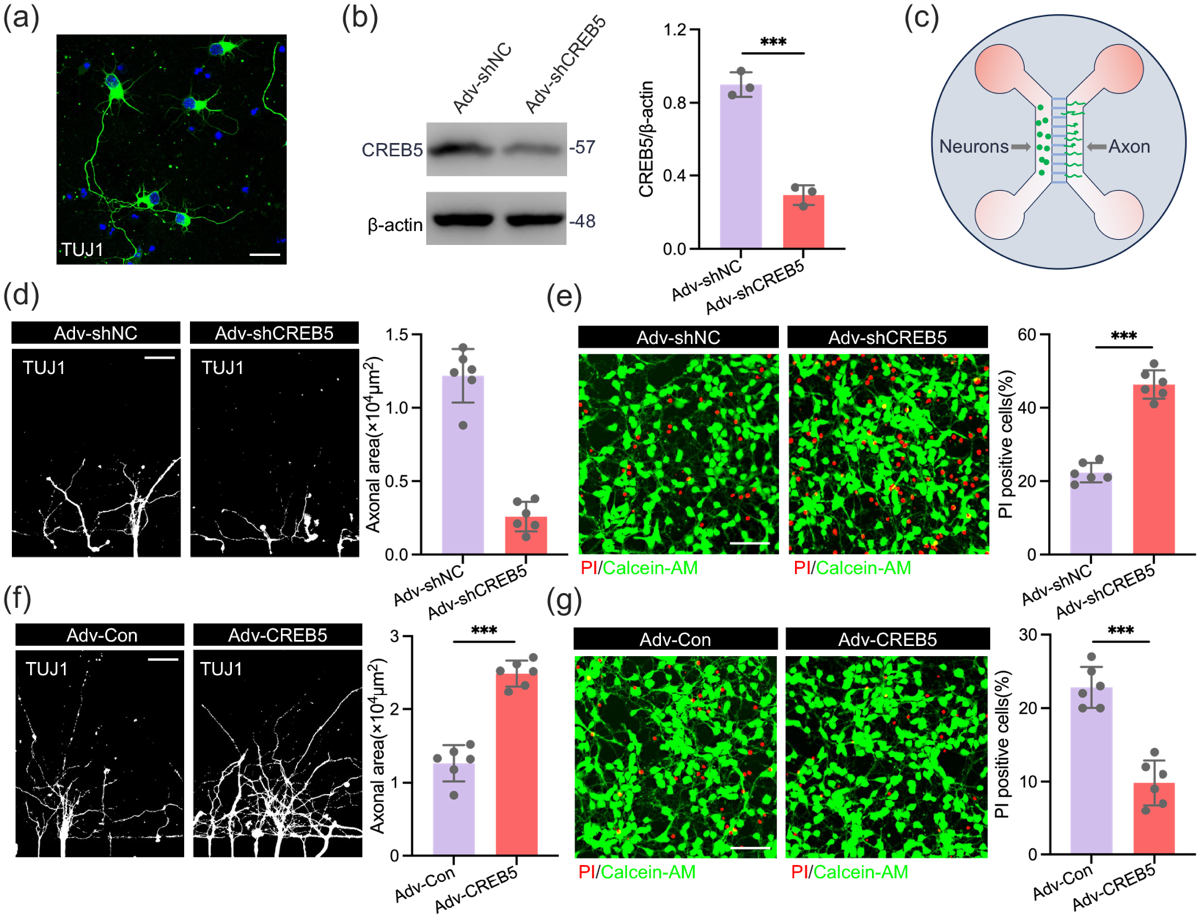
**Fig. S2 CREB5 is beneficial to the survival of neurons and axonal growth *in vitro*.** (a) β-tubulin III validates neuronal identity. Scale bar = 50 μm. (b) After knocking down CREB5 in neurons *in vitro*, the knockdown efficiency was verified by Western blot (n = 3). (c) Schematic diagram of the microfluidic device. (d) Axonal growth of neurons in the CREB5 knockdown group and control group, detected by anti-TUJ1 antibody labeling (n = 6). Scale bar = 100 μm. (e) Death ratios of neurons in the CREB5 knockdown group and control group, shown by PI/Calcein double staining (n = 6). Scale bar = 50 μm. (f) Axonal growth of neurons in the CREB5 overexpression group and control group, detected by anti-TUJ1 antibody labeling (n = 6). Scale bar = 100 μm. (g) Death ratios of neurons in the CREB5 overexpression group and control group, shown by PI/Calcein double staining (n = 6). Scale bar = 50 μm. Data were analyzed using the two-tailed unpaired Student's *t*-test (b, d, e, f and g). (***P < 0.001).


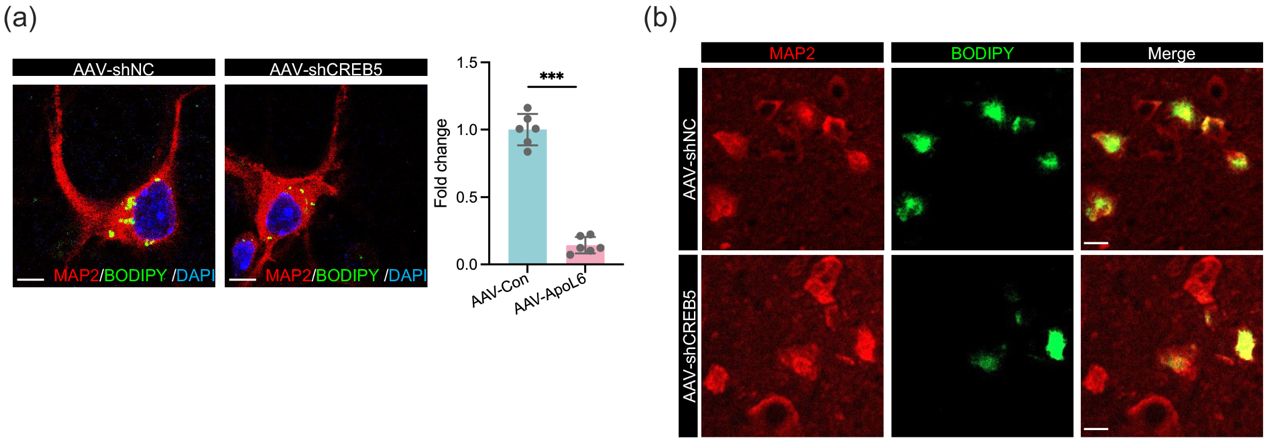
**Fig. S3 Knockdown of CREB5 promotes lipid droplet breakdown.** (a) Use BODIPY to detect lipid droplet levels in primary neurons from the control group and the CREB5 knockdown group. Scale bar = 5 μm (n = 6). (b) BODIPY and anti-MAP2 antibody were used to detect LD levels in neurons located 2 mm from the epicenter of SCI. Scale bar = 20 μm. Data were analyzed using the two-tailed unpaired Student's *t*-test (a). (***P < 0.001).


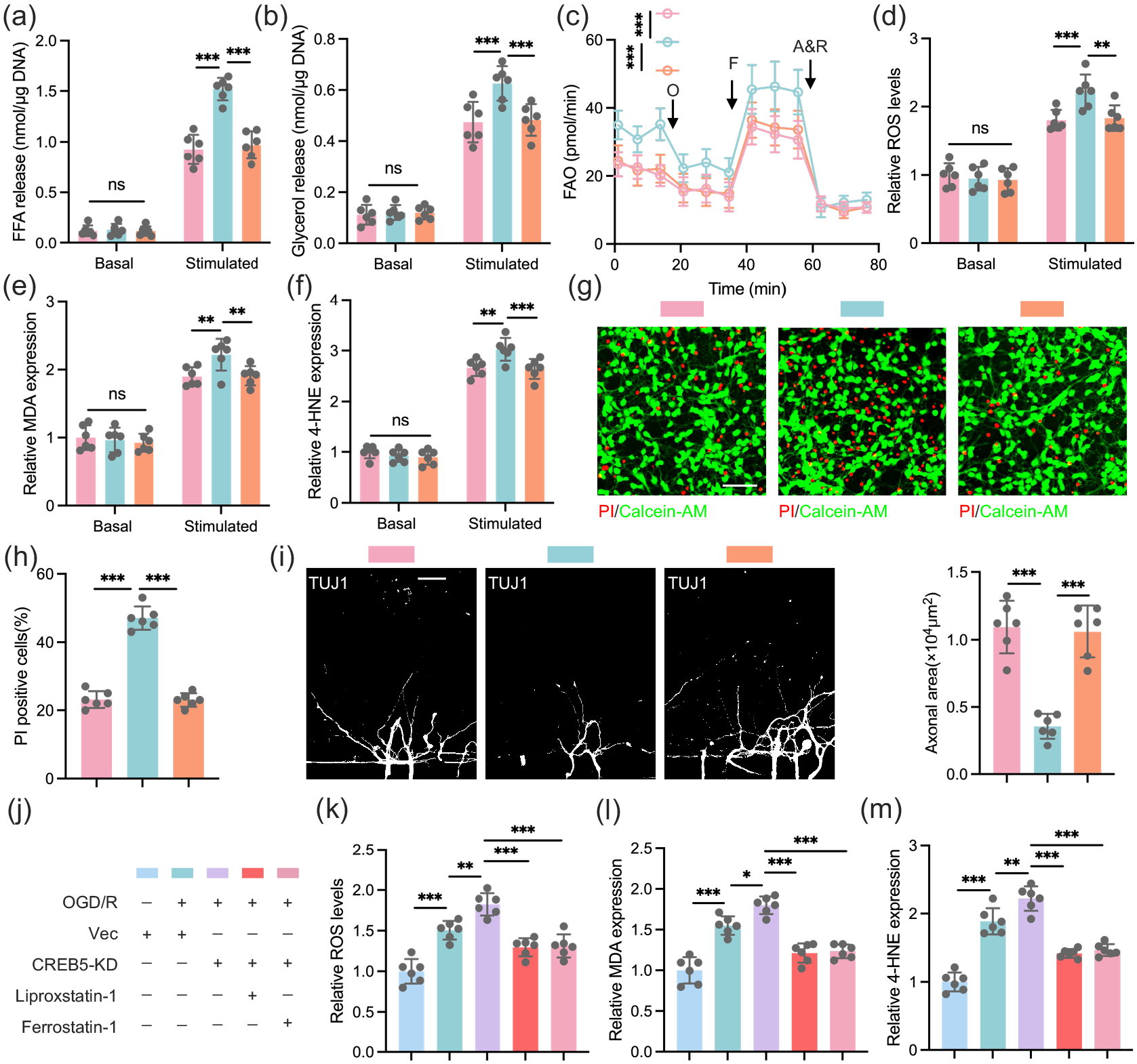
**Fig. S4 CREB5 inhibits neuronal ferroptosis by promoting ApoL6 expression.** (a, b) Detection results of FFA and glycerol release in neurons after isoproterenol treatment or control treatment, respectively (n = 6). (c) Detection results of neuronal FAO levels in experimental groups with different genotypes (n = 6). (d-f) Bar graphs respectively show the detection results of ROS, MDA and 4-HNE contents in neurons after isoproterenol treatment or NC treatment (n = 6). (g, h) PI/Calcein-AM double staining shows the death ratio of neurons with different genotypes after isoproterenol treatment and the quantitative statistics chart (n = 6). (i) After isoproterenol treatment of neurons, the axonal growth of different genotypes in the microfluidic device was observed by anti-TUJ1 antibody labeling (n = 6). (j) Schematic diagram of the grouping. (k-m) Bar chart showing the levels of ROS, MDA, and 4-HNE in neurons. Data were analyzed using one-way ANOVA (a, b, d, e, f, h, i, k, l and m) and two-way ANOVA (c) followed by post hoc Bonferroni correction. (*P < 0.05, **P < 0.01, ***P < 0.001).


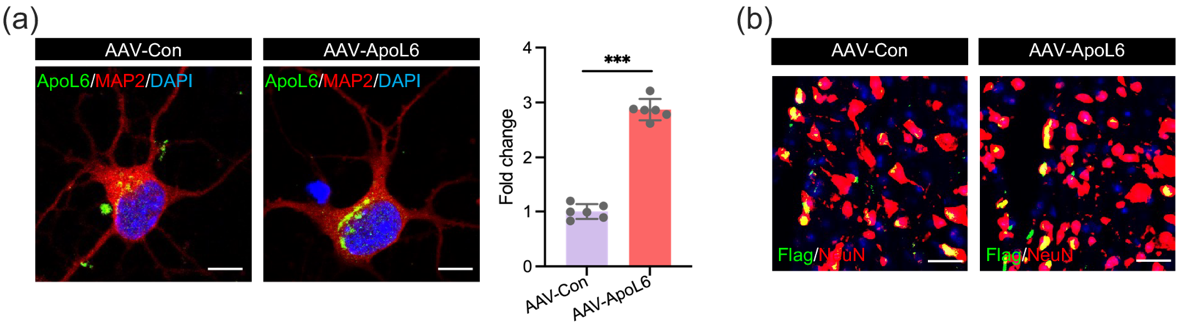


**Fig. S5 Overexpression of ApoL6 in neurons.** (a) Confocal images show the efficiency of ApoL6 overexpression in neurons (n = 6). Scale bar = 5 μm. (b) The transfection efficiency of the control group and the overexpressing ApoL6 group was detected by anti-Flag and anti-NeuN antibody labeling. Scale bar = 50 μm. Data were analyzed using the two-tailed unpaired Student's *t*-test (a). (***P < 0.001).

**Table S1 Nucleotide sequences of shRNA**

| ***Names*** | ***Species*** | ***Sequence (5′ to 3′)*** |
| --- | --- | --- |
| *Creb5*  Scramble | Mus musculus  Mus musculus | TTATGTGTTAACAACAACTGC  GATCCCGTTCTCCGAACGTGTCA  CGTTTCAAGAGAACGTGACACGTTC  GGAGAACTTTTTTG |
